# A Novel System for Simultaneous or Sequential Integration of Multiple Gene-Loading Vectors into a Defined Site of a Human Artificial Chromosome

**DOI:** 10.1371/journal.pone.0110404

**Published:** 2014-10-10

**Authors:** Teruhiko Suzuki, Yasuhiro Kazuki, Mitsuo Oshimura, Takahiko Hara

**Affiliations:** 1 Stem Cell Project, Tokyo Metropolitan Institute of Medical Science, Kamikitazawa, Setagaya-ku, Tokyo, Japan; 2 Department of Biomedical Science, Institute of Regenerative Medicine and Biofunction, Graduate School of Medical Science, Tottori University, Yonago, Tottori, Japan; 3 Chromosome Engineering Research Center, Tottori University, Yonago, Tottori, Japan; 4 Graduate School of Medical and Dental Sciences, Tokyo Medical and Dental University, Yushima, Bunkyo-ku, Tokyo, Japan; Imperial College London, United Kingdom

## Abstract

Human artificial chromosomes (HACs) are gene-delivery vectors suitable for introducing large DNA fragments into mammalian cells. Although a HAC theoretically incorporates multiple gene expression cassettes of unlimited DNA size, its application has been limited because the conventional gene-loading system accepts only one gene-loading vector (GLV) into a HAC. We report a novel method for the simultaneous or sequential integration of multiple GLVs into a HAC vector (designated as the SIM system) via combined usage of Cre, FLP, Bxb1, and φC31 recombinase/integrase. As a proof of principle, we first attempted simultaneous integration of three GLVs encoding *EGFP*, *Venus*, and *TdTomato* into a gene-loading site of a HAC in CHO cells. These cells successfully expressed all three fluorescent proteins. Furthermore, microcell-mediated transfer of HACs enabled the expression of those fluorescent proteins in recipient cells. We next demonstrated that GLVs could be introduced into a HAC one-by-one via reciprocal usage of recombinase/integrase. Lastly, we introduced a fourth GLV into a HAC after simultaneous integration of three GLVs by FLP-mediated DNA recombination. The SIM system expands the applicability of HAC vectors and is useful for various biomedical studies, including cell reprogramming.

## Introduction

Human artificial chromosomes (HACs) and mouse artificial chromosomes (MACs) are chromosomal gene-delivery vectors. They behave as independent extra chromosomes and are stably maintained in host cells. The biggest advantage of HACs/MACs over other DNA vectors such as P1 phage artificial chromosomes and bacterial artificial chromosomes (BACs) is that they basically have no size limitation for insert DNA. Employing the characteristic, human immunoglobulin locus and the dystrophin locus (2.4 Mb) have been successfully cloned on HACs and transferred to mouse cells [1]–[5].

The HAC vector was also utilized to generate induced pluripotent stem cells (iPSCs) as a vehicle for the four Yamanaka factors (*Oct4*, *Sox2*, *Klf4*, and *c-Myc*) [6], [7]. Two groups successfully induced iPSCs utilizing HACs and both showed that the introduction of multiple OCT4 expression units was important for the induction of iPSCs. These results indicate that optimal expression levels of reprogramming factor genes are essential for iPSC reprogramming. Since a HAC has no size limitation for insert DNA, it is possible to adjust expression levels of exogenous genes by changing the copy number of their expression units. Another advantage of the HAC expression system is that foreign genes on HACs can be removed by eliminating the HAC itself before therapeutic application [6], [8]–[11]. Fibroblasts or committed blood cells can be directly reprogrammed to hepatocytes, neurons, or hematopoietic stem-like cells by simultaneous overexpression of as many as eight genes [12]–[14]. HAC/MAC vectors would be also useful for such direct reprogramming owing to their superior gene-loading capacity.

HACs/MACs usually contain a site for accepting a gene-loading vector (GLV) to introduce transgenes. Among the various strategies for accurate introduction of GLVs to HACs/MACs [15]–[17], reconstitution of the hypoxanthine guanine phosphoribosyltransferase (*HPRT*) gene is one of the most popular selection principles [8], [18], [19]. In this case, HACs/MACs possess a loxP-flanking, phosphoglycerate kinase (PGK) promoter-driven 5′*HPRT* unit that consists of exon 1, 2, and the 5′ half of intron 2 of the *HPRT* gene (Fig. S1). On the other hand, GLV carries a loxP-flanking 3′*HPRT* unit consisting of the 3′ half of intron 2 and the remaining exons 3–9 of the *HPRT* gene. *HPRT* is reconstituted by Cre/loxP-mediated integration of GLV to the gene-loading site of HACs/MACs that makes HAC/MAC-bearing HPRT-deficient cells hypoxanthine-aminopterin-thymidine (HAT) resistant. Although this method is reliable and efficient, only one GLV can be introduced into HAC/MAC. Therefore, construction of an extremely large GLV is often required when introducing multiple gene expression cassettes in HAC/MAC [6], [20]. This procedure is labor-intensive and prone to cause unexpected errors.

To overcome this technical problem, researchers have been seeking an efficient method for introducing multiple genes into HAC/MAC [15], [16]. As a solution, we conceived the idea to utilize the splicing mechanism of the gene-loading site, which was originally designed to reconstitute the *HPRT* gene, for the simultaneous or sequential integration of multiple GLVs. We named this newly developed gene-loading system the SIM system for simultaneous/sequential integration of multiple gene-loading vectors. In this study, we demonstrate that the SIM system enables us to integrate three GLVs simultaneously into a defined gene-loading site of a HAC vector. We also show that GLVs can be sequentially introduced into a HAC by reciprocal usage of two selection markers based on the principle of gene trapping.

## Results

### Construction of GLVs for the SIM system

**Figure 1:**
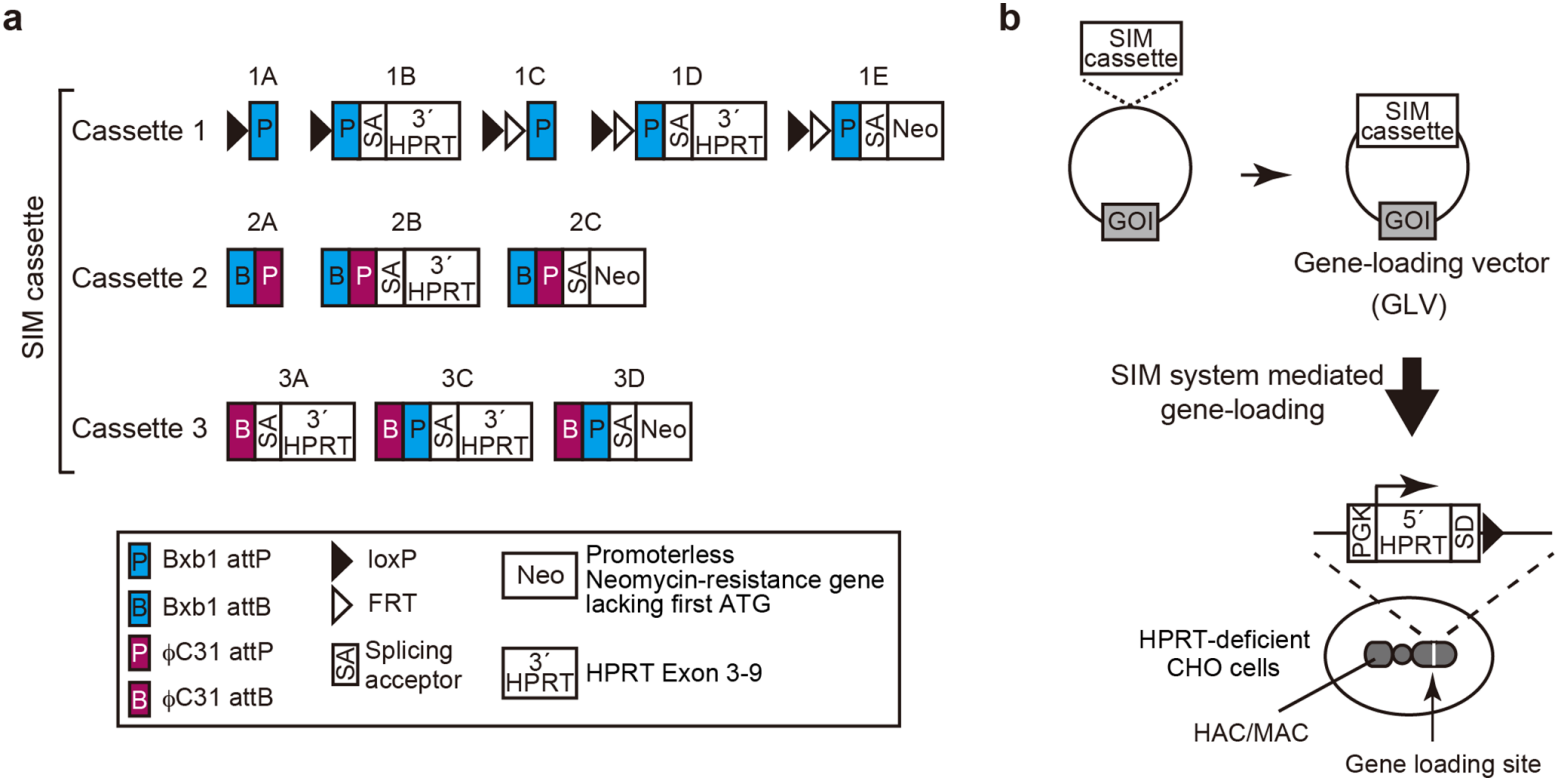
SIM cassettes and GLVs. (a) Schematic representation of SIM cassettes. SIM cassettes are classified into three groups based on the order of the use. Abbreviations and name of each cassette are shown. (b) A GLV was constructed by inserting a SIM cassette into a gene of interest (GOI)-encoding vector. For the sequential integration, first step is the introduction of a GLV having cassette 1 to the gene-loading site of the HAC using Cre recombinase. The cassette 1 contains Bxb1 attP sequence so that a GLV with cassette 2 can be integrated into the HAC as a second GLV by the use of Bxb1 integrase. φC31 attP sequence of the cassette 2 enables loading of the third GLV with cassette 3 using φC31 integrase. Afterward, alternative use of GVLs having cassette 2 or cassette 3 allows for sequential introduction of GLVs into the HAC. For the simultaneous integration, GLVs having cassette 1, 2 or 3 were loaded to the HAC simultaneously by using Cre, Bxb1 integrase, and φC31 integrase. For the combination of simultaneous and sequential integration of GLVs, cassette 1 containing FRT sequence was used. The FRT sequence makes it possible to integrate a fourth GLV having cassette 1 after the simultaneous integration of three GLVs. Fifth and more GLVs could be introduced in the same way based on the sequential loading procedure.

We first constructed a variety of modules called SIM cassettes, which consist of recognition sequences of Cre, FLP, Bxb1, and/or φC31 recombinase/integrase [21], a splicing acceptor sequence and/or drug resistance gene (Fig. 1a, Fig. S1). Cre and FLP are well-known tyrosine recombinases that catalyze reversible recombination reactions between two loxP and FLP recombinase target (FRT) sites, respectively [22]. Bxb1 and φC31 integrases, on the other hand, are serine integrases that catalyze irreversible recombination between attP and attB of each integrase [23]. Recombination between attP and attB generates their hybrid sequences attL and attR, which are no longer substrates for these integrases in the absence of a cofactor. We categorized all of these SIM cassettes into three groups (Cassette 1, 2, and 3), reflecting the order of use in this study. One of these SIM cassettes was inserted into a vector encoding a gene of interest (GOI) to construct a GLV (Fig. 1b).

### Simultaneous integration of three GLVs into a HAC

To explore the potential of the SIM system, we first attempted simultaneous integration of GLVs by transfecting three empty GLVs together with Cre, Bxb1, and φC31 recombinase/integrase expression vectors to *HPRT*-deficient CHO cells (CHO cells hereafter) carrying a HAC with a 5′*HPRT* unit as a gene-loading site. We consistently obtained six to eight HAT resistant CHO clones in three independent trials (Table 1). Only one HAT resistant clone was grown from HAC-free CHO cells in the three independent experiments, suggesting that the effect of background is negligible.

**Table 1:** Efficiency of simultaneous integration of GLVs.

Experiment	Cells	GLV1	GLV2	GLV3	# of colonies
#1	HAC/CHO	1C	2A	3A	8
#2	HAC/CHO	1C	2A	3A	7
#3	HAC/CHO	1C	2A	3A	6
#4	CHO	1C	2A	3A	0
#5	CHO	1C	2A	3A	0
#6	CHO	1C	2A	3A	1
#7	HAC/CHO	-	-	-	0

3×10^5^ target cells in a well of 12-well tissue culture plate were transfected with empty GLVs coding the indicated SIM cassettes together with expression vectors for Cre, Bxb1 integrase, and φC31 integrase. Cells were selected with HAT and resistant cells were stained with crystal violet to count the number of colonies.

**Figure 2:**
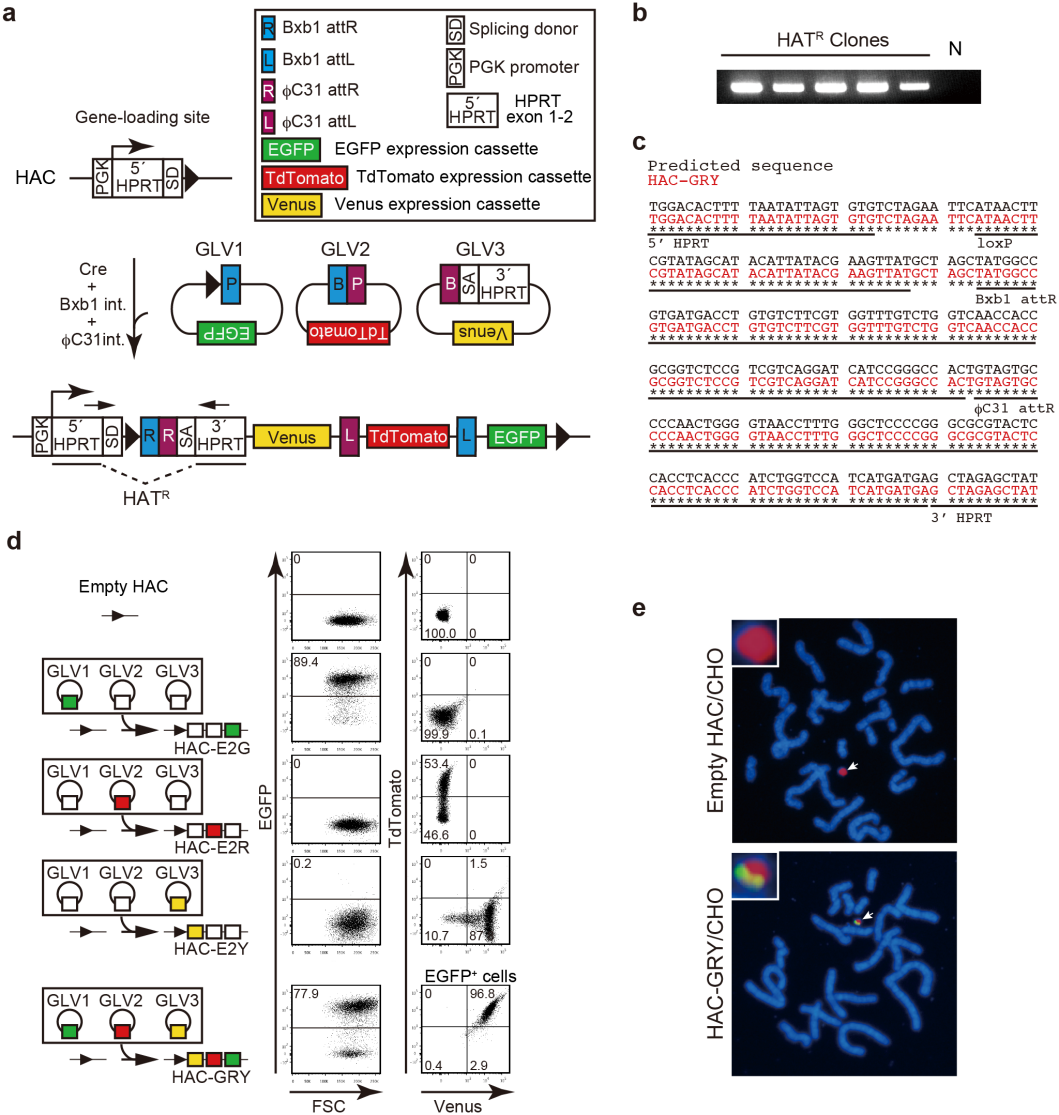
Simultaneous integration of GLVs into the HAC. (a) Schematic representation of the simultaneous integration of GLVs encoding *EGFP*, *TdTomato*, and *Venus* expression units into the HAC. Abbreviations are shown. Arrows indicate the position of PCR primers used for genotyping. (b) PCR analysis of the integration of GLVs into the HAC. Genomic DNA was prepared from HAT resistant CHO clones and CHO cells carrying an empty HAC for the negative control (N). (c) Sequencing analysis of the junctional region of reconstituted *HPRT*. Predicted sequence is shown in black. HAC-GRY sequence is shown in red. Sequence of each element is underlined. Asterisks indicate matched sequences. (d) GLVs encoding the *EGFP* expression unit (green box), *TdTomato* expression unit (red box), *Venus* expression unit (yellow box), or empty unit (white box) were introduced to CHO cells carrying the HAC. Structure of the GLVs-integrated HAC is depicted on the left. Abbreviations of the generated HACs are shown in the scheme. HAC-E2G, -E2R, and -E2Y were constructed as controls. The expression of fluorescent proteins was analyzed by flow cytometry. Percentages of positive cells in each cell population are shown in the dot plots. For a CHO clone carrying HAC-GRY, expression of TdTomato and Venus was analyzed in the EGFP^+^ population. Three fluorescent proteins were expressed simultaneously in CHO cells carrying HAC-GRY. (e) HAC (red) and transgenes (green) were detected by FISH using a digoxigenin-labeled human Cot-1 probe and biotin-labeled GLVs (GLV1, 2, and 3) probe, respectively. Arrows indicate HACs. Enlarged images of HACs are shown in insets.

To further evaluate the simultaneous integration system, we generated three GLVs (GLV1 with the *EGFP* gene, GLV2 with the *TdTomato* gene, and GLV3 with the *Venus* gene), and targeted to the gene-loading site of the HAC in CHO cells (Fig. 2a). Twenty-three clones were obtained from 6×10^5^ transfected cells that is comparable to the loading efficiency of empty GLVs. We randomly chose five HAT resistant clones and confirmed that all of them possessed the reconstituted *HPRT* gene (Fig. 2b). Furthermore, using sequencing analysis, we found loxP, Bxb1 attR, and φC31 attR sequences between 5′*HPRT* and 3′*HPRT* (Fig. 2c). Consistent with these results, flow cytometric analysis revealed concomitant expression of the three fluorescent proteins (Fig. 2d). On the other hand, when we transfected one of the GLVs together with two empty GLVs, we obtained HAT-resistant CHO clones emitting the predicted single fluorescence.

**Figure 3:**
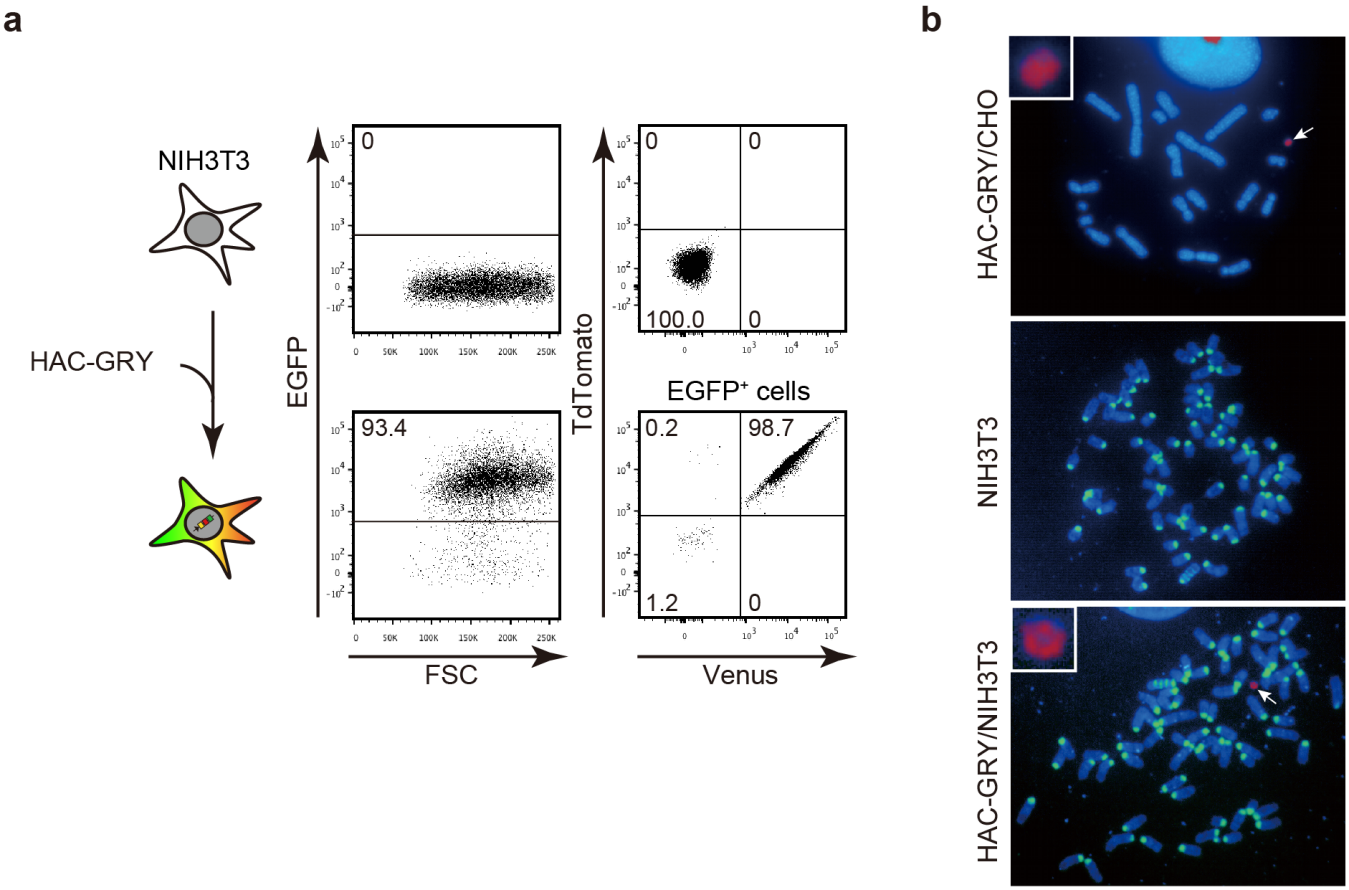
Transfer of HAC-GRY. (a) HAC-GRY was transferred into NIH3T3 cells by microcell-mediated chromosome transfer and the expression of fluorescent proteins was analyzed by flow cytometry. Top panels, parental NIH3T3 cells; bottom panels, NIH3T3 cell carrying HAC-GRY. For NIH3T3 clones carrying HAC-GRY, the expression of TdTomato and Venus was analyzed in the EGFP^+^ population. (b) HAC (red) and mouse chromosomes (green) were detected by FISH using a digoxigenin-labeled human Cot-1 probe and a biotin-labeled mouse major satellite probe, respectively. Arrows indicate HACs. Enlarged images of HACs are shown in insets.

We confirmed the presence of GLVs-derived sequence (Fig. 2e, green) on the HAC (Fig. 2e, red) by fluorescence *in* *situ* hybridization (FISH). The recombinant HAC was successfully transferred into NIH3T3 cells by microcell-mediated chromosome transfer and recipient NIH3T3 cells acquired the expression of three fluorescent proteins as expected (Fig. 3a). FISH analysis of the HAC-containing NIH3T3 cells proved that all chromosomes except the HAC were of mouse origin (Fig. 3b). Therefore, all of the fluorescent genes localized on the HAC; there was no cross contamination of CHO-derived hamster chromosomes. Taken together, these results demonstrated that the SIM system works for the simultaneous integration of three GLVs to a defined site of the HAC in mammalian cells.

### Sequential integration of GLVs into a HAC

**Figure 4:**
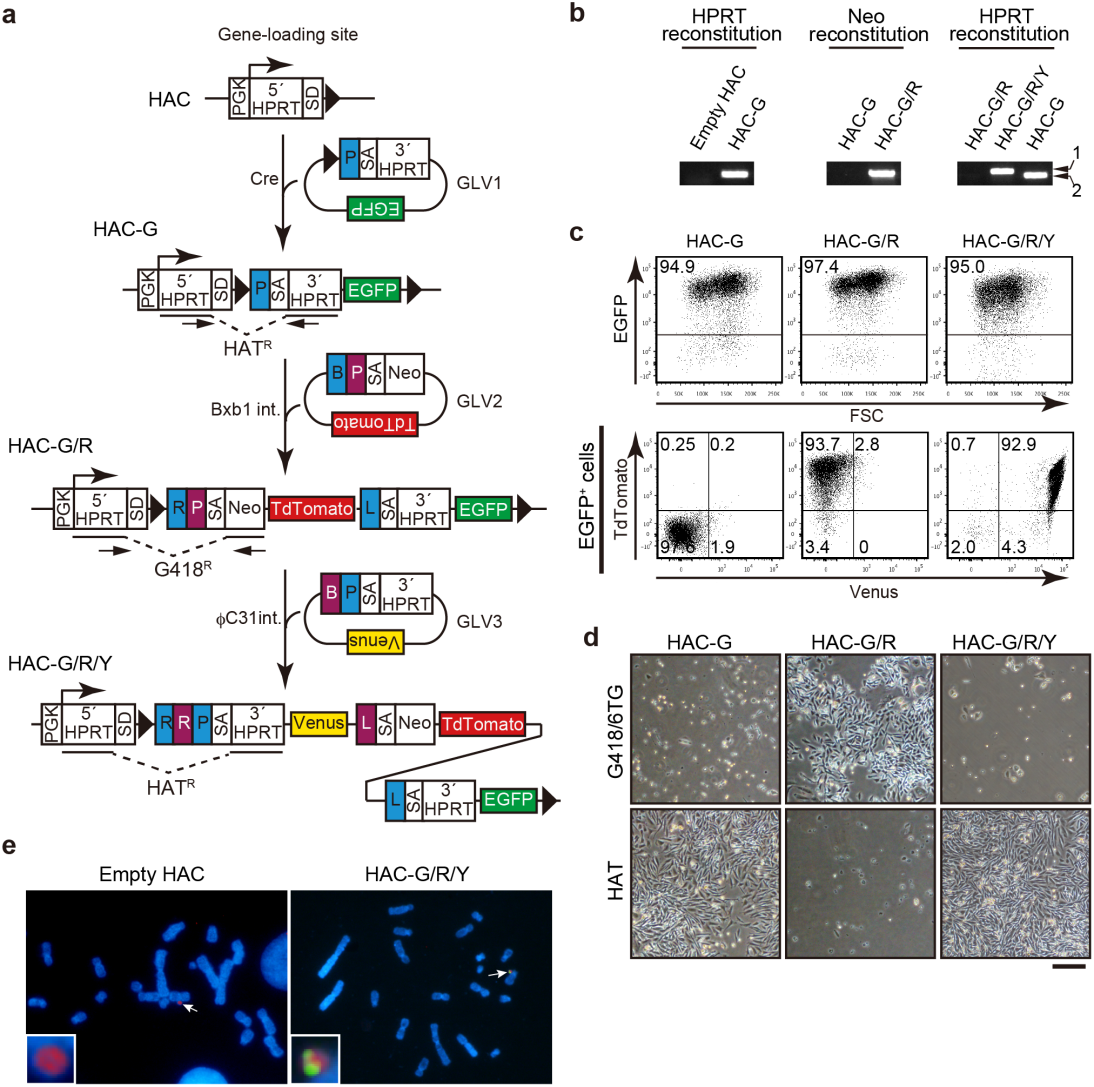
Sequential integration of GLVs to the HAC. (a) Schematic representation of the sequential integration of GLVs to the HAC. PCR primers to detect reconstituted selection marker genes are depicted by arrows. (b) Reconstitution of *HPRT* or *5′HPRT-Neo* was analyzed by PCR. Note the amplicon from the HAC carrying GLV1, 2, and 3 (HAC-G/R/Y, arrow 1) was slightly larger than that of HAC-G due to attR sequences generated by the second and third round of the integration reaction. (c) The expression of fluorescent proteins was analyzed by flow cytometry. Percentages of positive cells in each population are shown in the dot plots. Expression of TdTomato and Venus was analyzed in the EGFP^+^ population. (d) CHO cells carrying the indicated HAC were cultured in medium supplemented with HAT or 800 µg/ml G418, HT, and 10 µg/ml 6TG. Scale bar, 200 µm. (e) HAC (red) and transgenes (green) were detected by FISH using a digoxigenin-labeled human Cot-1 probe and a biotin-labeled GLVs (GLV1, 2, and 3) probe, respectively. Arrows indicate HACs. Enlarged images of HACs are shown in insets.

We also tested the sequential loading of GLV1 (encoding *EGFP*), GLV2 (encoding *TdTomato*), and GLV3 (encoding *Venus*) by the SIM system. The principle of the method is illustrated schematically in Figure 4a. First, GLV1 was introduced into the HAC by Cre-mediated recombination. The resultant HAT-resistant CHO clone carried reconstituted *HPRT* and expressed the EGFP protein (Fig. 4b and c, HAC-G).

Next, we transfected GLV2 in combination with the Bxb1 integrase expression vector in the CHO cells carrying HAC-G. A recombination event triggered the expression of a 5′HPRT-Neo fusion protein, thereby causing cells to become G418 resistant (Fig. 4a, HAC-G/R). We confirmed reconstitution of 5′*HPRT-Neo* and expression of both EGFP and TdTomato (Fig. 4b and c, HAC-G/R).

As integration of GLV2 trapped promoter activity of *HPRT* reconstituted by GLV1, we were able to select CHO cells carrying a GLV3-integrated HAC with HAT again. *HPRT* was reconstituted in these CHO cells (Fig. 4b, HAC-G/R/Y). They simultaneously expressed EGFP, TdTomato, and Venus (Fig. 4c). We confirmed reciprocal switching of drug resistance between HAT and G418/6-thioguanine (6TG) during the process of stepwise gene loading (Fig. 4d). Lastly, we detected the integrated GLVs on HAC-G/R/Y by FISH (Fig. 4e). These data provide a proof of principle for the sequential loading of GLVs to a HAC by the SIM system. Theoretically, it is possible to load an unlimited number of genes by alternative operations of Bxb1 integrase-G418/6TG selection and φC31 integrase-HAT selection (Fig. S1).

### Combination of the simultaneous and sequential integration of GLVs

**Figure 5:**
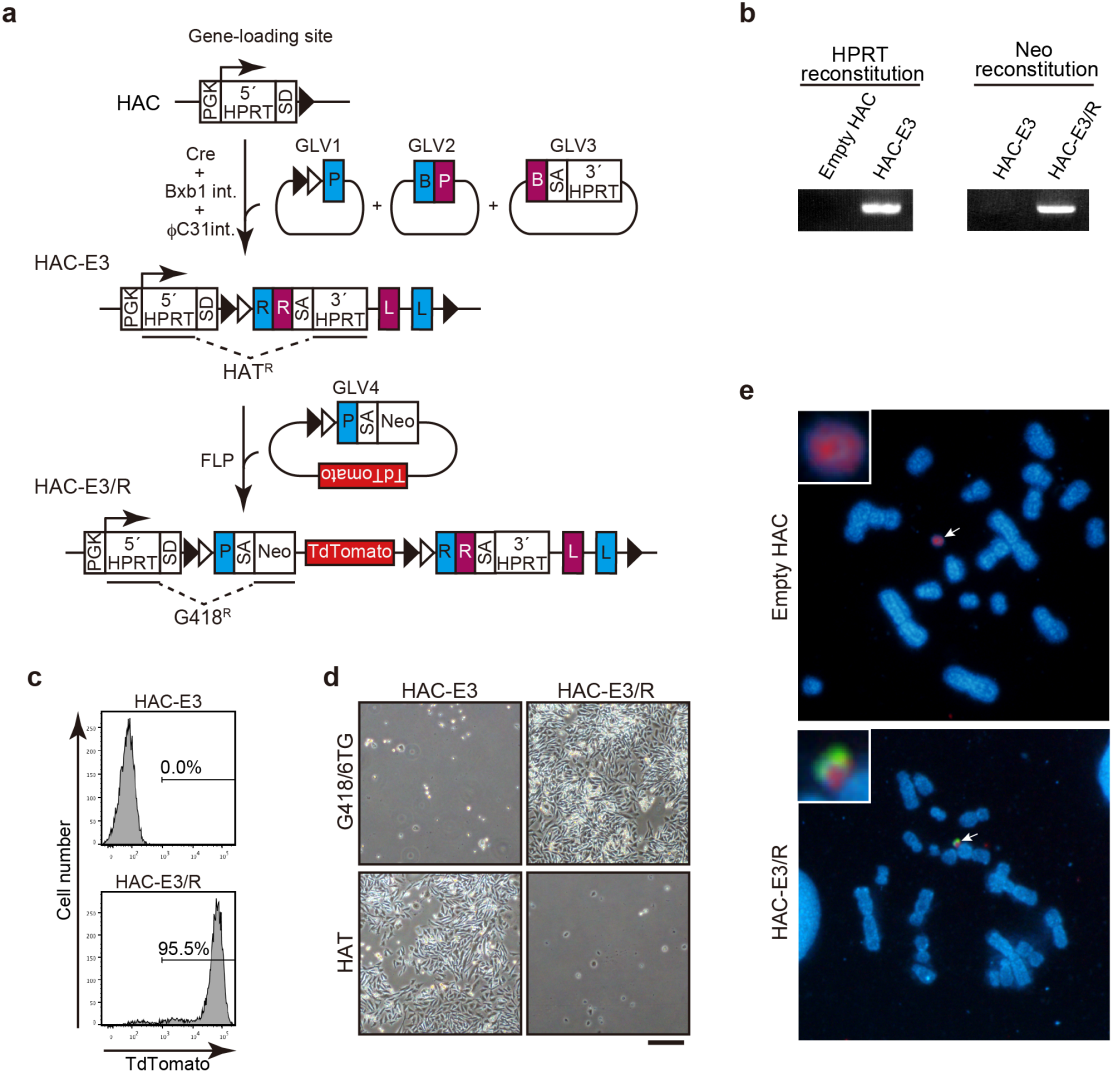
Combination of simultaneous and sequential integration of GLVs to the HAC. (a) Schematic representation of simultaneous integration followed by sequential integration of GLVs. (b) PCR analysis of the integration of GLVs to the HAC. (c) The expression of TdTomato was analyzed by flow cytometry. (d) CHO cells carrying the indicated HAC were cultured in medium supplemented with HAT or 800 µg/ml G418, HT, and 10 µg/ml 6TG. Scale bar, 200 µm. (e) HAC (red) and transgenes (green) were detected by FISH using a digoxigenin-labeled human Cot-1 probe and a biotin-labeled *TdTomato* probe, respectively. Arrows indicate HACs. Enlarged images of HACs are shown in insets.

Although the sequential integration system can load unlimited GLVs into a HAC, cell cloning is necessary for each step. On the other hand, the simultaneous integration system is convenient for quickly loading up to three GLVs. We combined both protocols to develop an efficient method for integration of four or more GLVs into a HAC. For this purpose, FRT sequence was introduced into a SIM cassette 1 series (Fig. 1a). After loading empty GLV1, 2, and 3 by the simultaneous integration system, we transfected the fourth GLV carrying SIM cassette 1E and *TdTomato* together with the FLP expression vector (Fig. 5a). We successfully obtained CHO cells carrying quadruple GLVs-integrated HACs by G418 selection. They possessed reconstituted 5′*HPRT-Neo* (Fig. 5b, HAC-E3/R), displayed TdTomato fluorescence (Fig. 5c), and became HAT-sensitive (Fig. 5d). We also confirmed the presence of the *TdTomato* gene on the HAC in CHO cells (Fig. 5e). These data proved that the combination protocol works for the efficient introduction of four or more GLVs into the HAC.

## Discussion

We reported the simultaneous integration of up to three GLVs to a gene-loading site of a HAC via the SIM system. To our knowledge, this is the first system developed for the simultaneous introduction of multiple GLVs to a defined site in mammalian cells. The SIM system facilitated simultaneous site-directed integration of multiple GLVs into the genome, a process previously presumed to be difficult [24]. According to previous reports, intermolecular integration between a plasmid vector and an episomal vector is significantly more efficient than between a plasmid vector and chromosome, probably due to their accessibility [24], [25]. Thus, we speculate that the integration reaction initially took place among three GLVs and then a unified GLV was integrated into a gene-loading site. In this case, the whole procedure requires only single rate-limiting integration reaction into the chromosomal DNA. The SIM system accurately integrated multiple GLVs on the HAC (Fig. 2b and c) due to the faithful recombination reactions of the recombinase/integrases. Nevertheless, intactness of the introduced genes still needs to be checked. In fact, we found an EGFP-negative population in HAC-GRY cells (Fig. 2d) expressed none of fluorescent proteins and the fluorescent genes were detected neither on the HAC nor the host chromosomes (data not shown). Loss of the insert DNA could be a result of spontaneous recombination in the HAC during culture. While such aberrant HAC can be eliminated by cell cloning (Fig. 3), precise validation of the transgenes should be performed before utilization of the gene-loaded HAC.

We also demonstrated that the SIM system enabled us to introduce GLVs into a HAC one by one. A few laboratories have developed related methods for the integration of multiple GLVs. Previous study showed that introduction of multiple transgenes into a HAC was possible by sequential homologous recombination in DT40 cells [18]. However, this procedure required laborious construction of gene targeting vectors for each transgenes. Yamaguchi *et al*. [16] reported more convenient gene-loading system, the multi-integrase (MI) system, which utilizes different kinds of recombinases/integrases for introducing up to five GLVs to a gene-loading platform. While the MI system enabled effective loading of GLVs, it requires as many selection markers as GLVs to be loaded. Another system solved this limitation by employing Cre to delete the selection marker gene after use [15]. A disadvantage of this system is that it requires an extra round of cloning to remove the selection marker gene before loading the next GLV. Furthermore, one loxP site remains after excision of the selection marker gene and can cause a problem when repeating Cre-mediated excision of selection marker genes for sequential GLV loading. The SIM system requires only two selection marker genes for multiple GLV loading and does not require deletion of selection marker genes, since recombination of GLV switches the expression of selection marker genes by trapping promoter activity of the previous drug resistance gene. These properties of the SIM system are apparently superior to the previous gene-loading methods.

We showed that the combination of simultaneous and sequential integration is useful for efficient introduction of four or more GLVs on a HAC. As only one GLV can be introduced on a HAC by the conventional gene-loading procedure, an extremely large GLV has to be constructed when multiple expression units are loaded [6], [20]. By using the SIM system, each gene expression unit can be separately introduced by multiple GLVs. In addition, this system also allows us to modify the HAC even after the integration of multiple GLVs.

**Figure 6:**
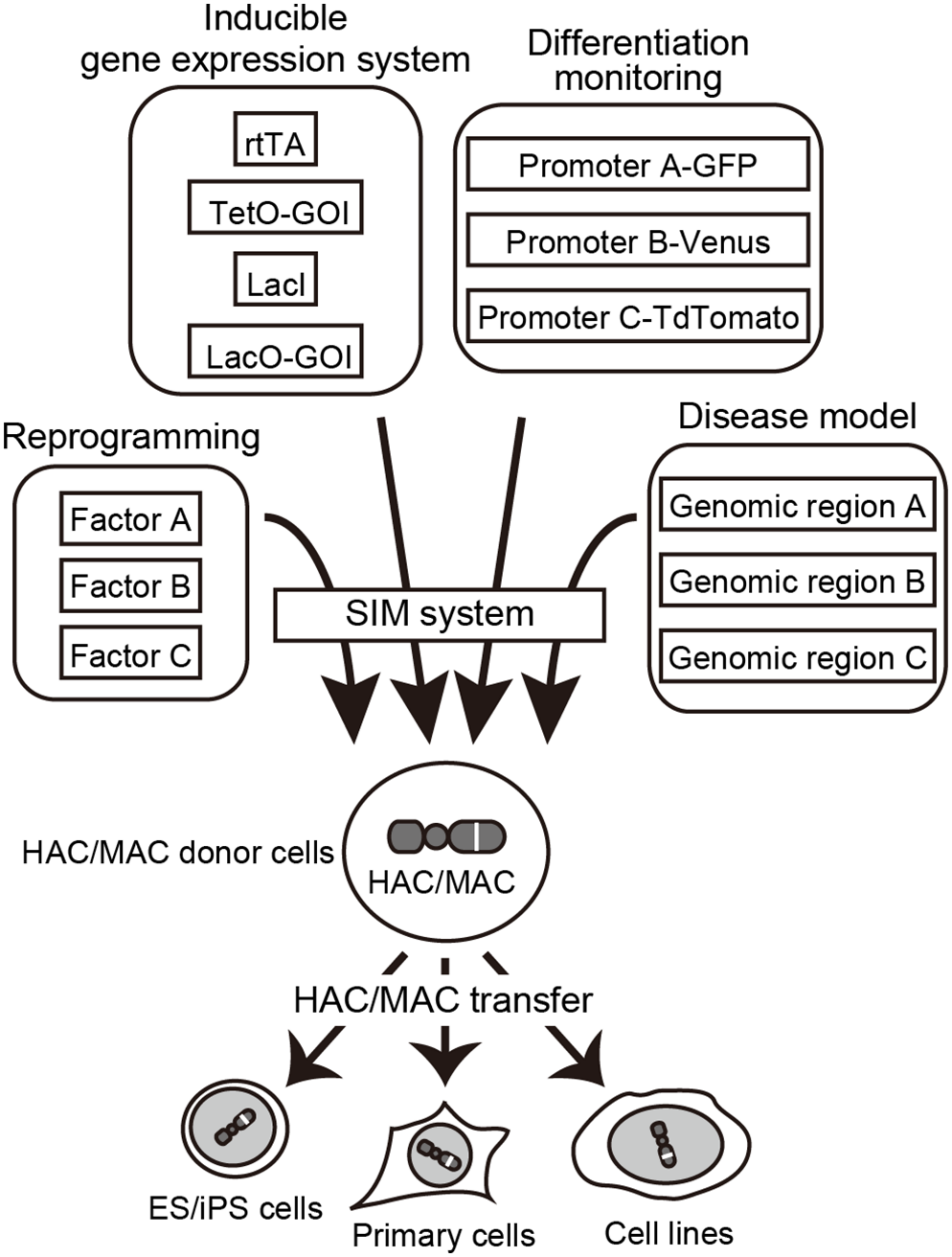
Expected application of the SIM system. Multiple gene expression units can be separately cloned to multiple GLVs and loaded into HACs/MACs by the SIM system. This enables efficient construction of HACs/MACs carrying multiple gene expression units, for example, cell reprogramming factors, components of Tet and/or Lac inducible gene expression systems, surrogate fluorescent reporters, and disease-related genomic fragments. These HACs/MACs can be easily transferred from donor CHO cells to recipient cells, including iPSCs, immortalized cell lines, and primary cells.

Since multiple gene expression units can be efficiently introduced into a HAC/MAC by the aid of SIM system, expanded applications of the technology are possible (Fig. 6). For example, a HAC/MAC carrying the optimum copy number of reprogramming factor genes could be efficiently constructed to generate uniform and high quality reprogrammed cells. It is worth noting that the HAC we used in this study is the conditionally removable type of HAC [26]. This property provides another advantage for removing reprogramming factors in the HAC after the cellular reaction. It could also be applicable to load multiple fluorescent gene cassettes under control of different lineage-specific promoters to trace differentiation status by multi-color live imaging. As a recent application of the HAC technology, a BAC clone covering genomic DNA derived from human chromosome 21 was introduced into a HAC to analyze gene function for a pathological phenotype of Down syndrome in mice [27]. Although we demonstrated the efficient introduction of multiple GLVs into the HAC in this study, plasmid-based vectors have size limitation for insert DNA. If the SIM system is employed to load multiple BACs, it could be possible to examine multicomponent effects of chromosome 21 genes on its pathology by transferring several hundred kilobases to megabase-size of DNA to a HAC. While loading efficiency of BAC-based vectors may be lower than that of plasmid-based GLVs, loading of multiple BACs is presumably possible because Bxb1 and φC31 integrases are known to mediate recombination reactions very efficiently [16].

The SIM system is a universally applicable gene-loading procedure to any existing HAC/MAC that utilizes the splicing-based systems for reconstituting selection marker genes. Moreover, it can be used to integrate multiple GLVs to a defined site of chromosomal DNA in the cells if a gene-loading site for a SIM cassette is introduced into human/mouse genomes by CRISPR/Cas9-mediated genome editing. This study provides a solid basis for these future applications.

## Materials and Methods

### Construction of vectors for the SIM system

DNA fragments of loxP, FRT, φC31 attP, φC31 attB, Bxb1 attP, Bxb1 attB, and adenovirus genome-derived splicing acceptor sequence were synthesized (GenScript) based on previously reported sequences [16], [28]. Sequences of these elements are listed in Table S1. All the SIM cassettes were cloned in a pUC57-based cloning vector (GenScript). φC31 integrase and Bxb1 integrase expression vectors were a kind gift from Dr. T. Ohbayashi (Tottori University) [16]. For construction of GLVs, an appropriate SIM cassette was inserted into a vector encoding a GOI via conventional restriction digestion and ligation. For the fluorescent gene expression units, PGK, EF1, or CAG promoter-driven *EGFP*, *TdTomato*, and *Venus* expression units were used. These expression units were encompassed by chicken HS4 insulator sequence [6].

### Cell culture

For the gene-loading experiments, we used *HPRT*-deficient CHO cells (JCRB0218, JCRB Cell Bank) carrying the conditionally removable HAC (named BHI 1-38) that has one copy of the 5′*HPRT* unit with known overall structure [8], [29]. CHO cells carrying the HAC were cultured as described previously [8]. NIH3T3 cells (CRL-1658, ATCC) were cultured in Dulbecco’s modified Eagle’s medium supplemented with 10% fetal bovine serum (Life Technologies) and penicillin/streptomycin (Sigma-Aldrich). To analyze reciprocal switching of the expression of drug resistance genes by the sequential integration of GLVs, 5×10^4^ cells were seeded per well of 6-well plate in medium supplemented with HAT Media Supplement (Sigma-Aldrich) or G418 (800 µg/ml, Life Technologies), 6TG (10 µg/ml, Sigma-Aldrich), and Hypoxanthine-Thymidine (HT) Media Supplement (Sigma-Aldrich). Cells were cultured for 4 days and photographed with an inverted microscope IX71 equipped with a CCD camera DP72 (Olympus).

### Loading of GLVs by the SIM system

For the simultaneous integration of GLVs, GLVs 1 to 3 together with Cre, Bxb1 integrase, and φC31 integrase expression vectors were co-transfected into CHO cells carrying the HAC using Lipofectamine LTX (Life Technologies) according to the manufacturer’s protocol. Briefly, 1,750 ng total of GLV 1, 2, and 3 in a molar ratio of 1∶2∶3 together with 250 ng each of recombinase/integrase expression vector were transfected to 6×10^5^ target cells in a well of 6-well plate. For the sequential integration of GLVs, a GLV together with the corresponding recombinase/integrase expression vector were co-transfected to target cells. For transfection in a well of 6-well plate, 2,250 ng of a GLV and 250 ng of a recombinase/integrase expression vector were used. Transfected cells were replated on the next day and HAT or 800 µg/ml G418 and HT were added to the medium two days after transfection. For cells under selection with G418, 10 µg/ml 6TG was added to the medium 3 days after starting selection for further elimination of cells expressing HPRT. Primers to confirm reconstitution of the *HPRT* gene were as follows: *HPRT* Fw, 5′-TGGAGGCCATAAACAAGAAGAC-3′; *HPRT* Rv, 5′-CCCCTTGACCCAGAAATTCCA-3′. To confirm reconstitution of the *Neo* gene, a primer (5′-CGCCTTGAGCCTGGCGAACA-3′) was used in combination with the *HPRT* Fw primer. To analyze the sequence of the junctional region of the reconstituted *HPRT* gene, the sequence was amplified by PCR with the primer pair to confirm *HPRT* reconstitution using template genomic DNA prepared from a HAT resistant CHO clone carrying HAC-GRY. PCR amplified sequence was analyzed by a 3130xL Genetic Analyzer (Applied Biosystems). HAC-GRY was transferred to NIH3T3 by a slightly modified method of microcell-mediated chromosome transfer [4] (Suzuki T. et al., manuscript in preparation).

### Flow cytometry

Expression of fluorescent proteins was analyzed by FACSAria (BD Bioscience). EGFP and Venus were discriminated using specific optical filter sets as previously described [30]. The data were processed with FlowJo vX.0.7 (Tree Star).

### FISH analysis

FISH analysis was performed on either fixed metaphase or interphase nuclei, as described previously with slight modification [4]. Briefly, digoxigenin-labeled human Cot-1 (Life Technologies) and specific biotin-labeled probes were prepared with Nick Translation Mix (Roche). Template DNAs for mouse major satellite sequence and *TdTomato* were prepared by PCR amplification. For mouse major satellite sequence, two copies of mouse major satellite sequence cloned into the pGEMT Easy Vector (Promega) were used as a PCR template. After hybridization, the TSA Biotin Kit (PerkinElmer) was used to enhance the signal of specific probes but not the major satellite sequence. Samples were counterstained with 4′,6-diamidino-2-phenylindole (DAPI) to visualize genomic DNA.

## Supporting Information

Figure S1 **Overview of the SIM system.** (a) Cre or FRT recombinase-mediated integration reaction. Note that the reaction is reversible. (b) φC31 or Bxb1 integrase-mediated integration reaction. Note that the reaction is irreversible. (c) Schematic representation of the SIM-mediated simultaneous or sequential integration of GLVs to the gene-loading site of a HAC/MAC. The conventional gene-loading system is also shown.
(TIF)

Table S1 **Synthetic DNA sequences.**
(DOCX)
