# Supplementary figures and images for: A Novel System for Simultaneous or Sequential Integration of Multiple Gene-Loading Vectors into a Defined Site of a Human Artificial Chromosome
Source: PLoS One. 2014 Oct 10;9(10):e110404. doi: 10.1371/journal.pone.0110404 (PMC4193884; doi:10.1371/journal.pone.0110404)

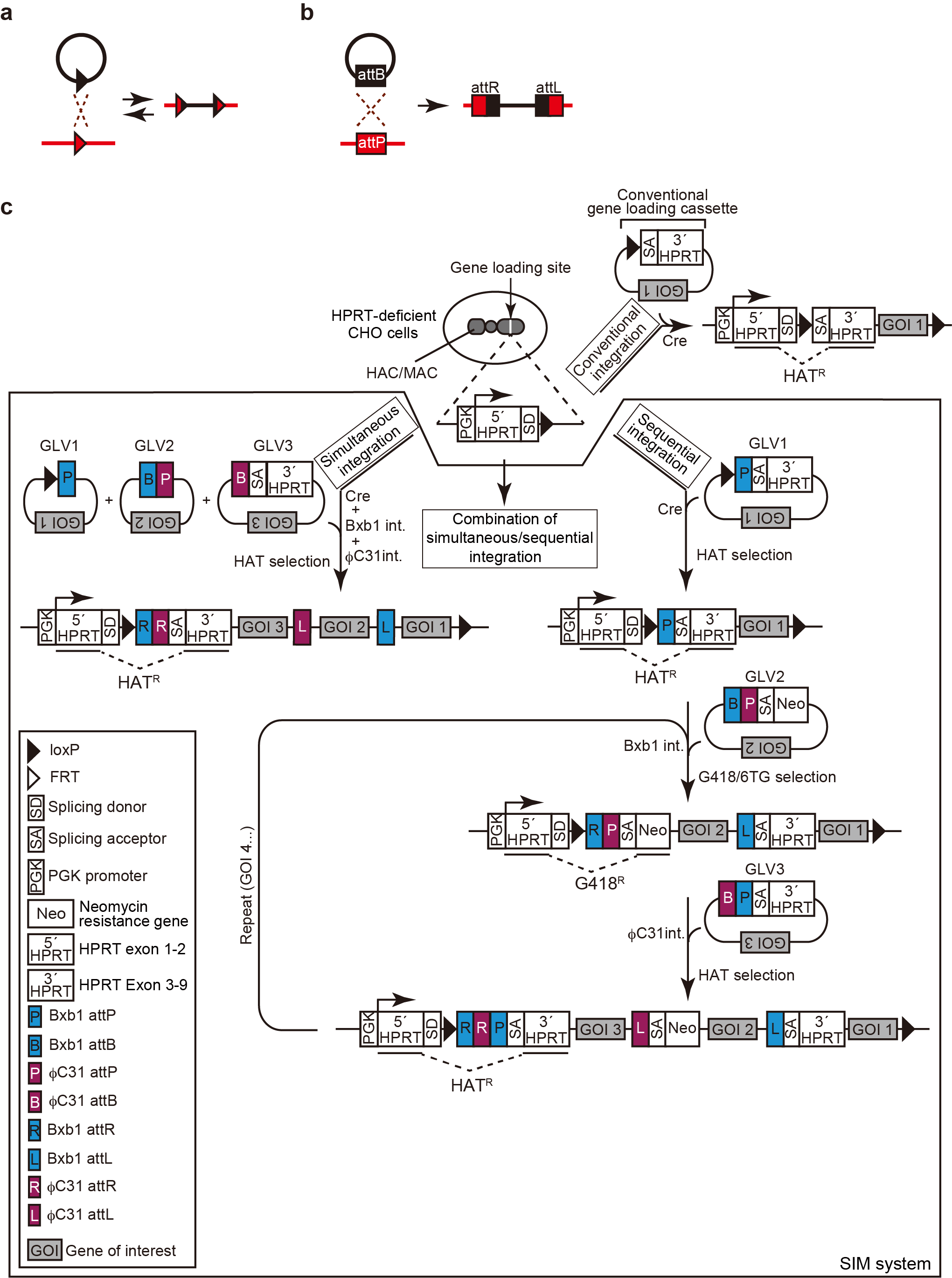

Supplement: Figure S1 — Overview of the SIM system. (a) Cre or FRT recombinase-mediated integration reaction. Note that the reaction is reversible. (b) φC31 or Bxb1 integrase-mediated integration reaction. Note that the reaction is irreversible. (c) Schematic representation of the SIM-mediated simultaneous or sequential integration of GLVs to the gene-loading site of a HAC/MAC. The conventional gene-loading system is also shown. (TIF) [file pone.0110404.s001.tif]
