# Supplementary material for: A Novel System for Simultaneous or Sequential Integration of Multiple Gene-Loading Vectors into a Defined Site of a Human Artificial Chromosome
Source: PLoS One. 2014 Oct 10;9(10):e110404. doi: 10.1371/journal.pone.0110404 (PMC4193884; doi:10.1371/journal.pone.0110404)
Supplement: Table S1 — Synthetic DNA sequences. (DOCX) [file pone.0110404.s002.docx]

**Table S1.** Synthetic DNA sequences.

| **Element** | **Sequence** |
| --- | --- |
| loxP | 5´-ATAACTTCGTATAGCATACATTATACGAAGTTAT-3´ |
| FRT | 5´-GAAGTTCCTATTCCGAAGTTCCTATTCTCTAGAAAGTATAGGAACTTC-3´ |
| φC31 attP | 5´-GTAGTGCCCCAACTGGGGTAACCTTTGAGTTCTCTCAGTTGGGGGCGTAGGGTC-3´ |
| φC31 attB | 5´-GATGTAGGTCACGGTCTCGAAGCCGCGGTGCGGGTGCCAGGGCGTGCCCTTGGGCTCCCCGGGCGCGTACTCCACCTCACCCATCTGGTCCATCATGATGA-3´ |
| Bxb1 attP | 5´-TATGGCCGTGATGACCTGTGTCTTCGTGGTTTGTCTGGTCAACCACCGCGGTCTCAGTGGTGTACGGTACAAACCCA-3´ |
| Bxb1 attB | 5´-TGGCCGTGGCCGTGCTCGTCCTCGTCGGCCGGCTTGTCGACGACGGCGGTCTCCGTCGTCAGGATCATCCGGGCCAC-3´ |
| Splicing acceptor sequence | 5´-CGTGACCTGCACGTCTAGGGCGCAGTAGTCCAGGGTTTCCTTGATGATGTCATACTTATCCTGTCCCTTTTTTTTCCACAGCTCGCGGTTGAGGACAAACTCTTCGCGGTCTTTCCAA-3´ |
